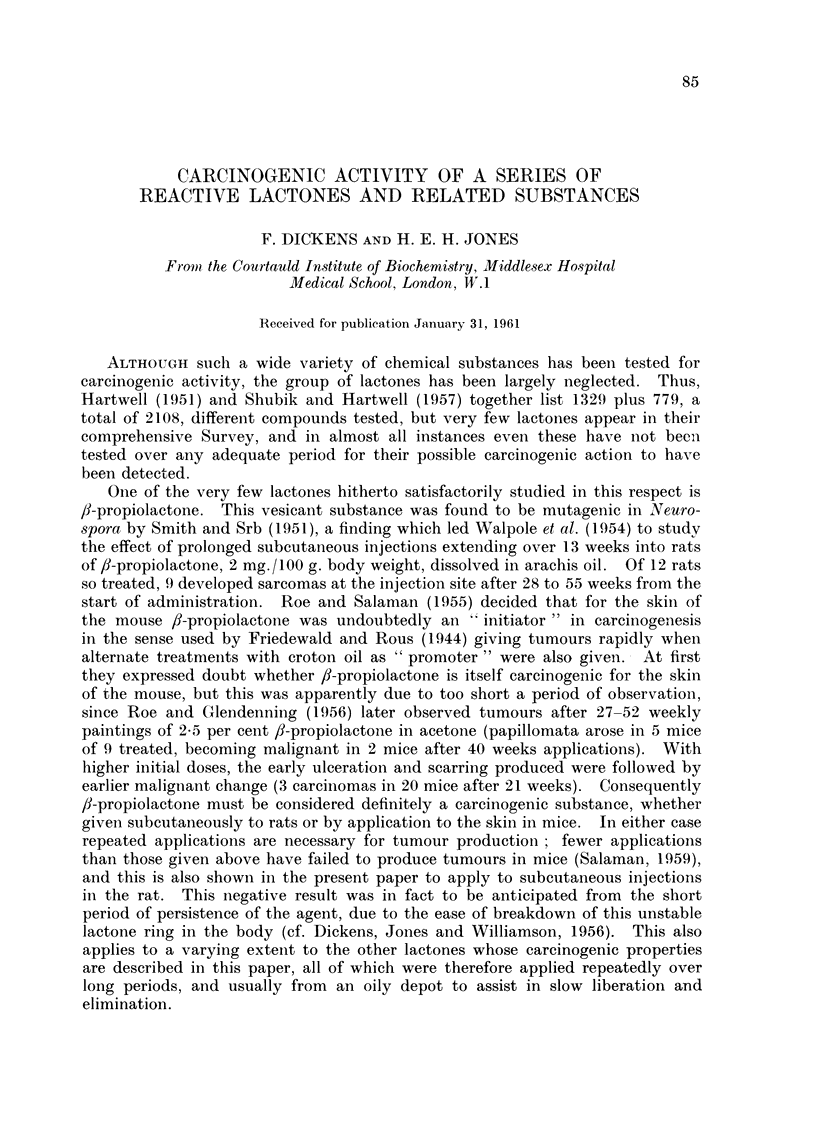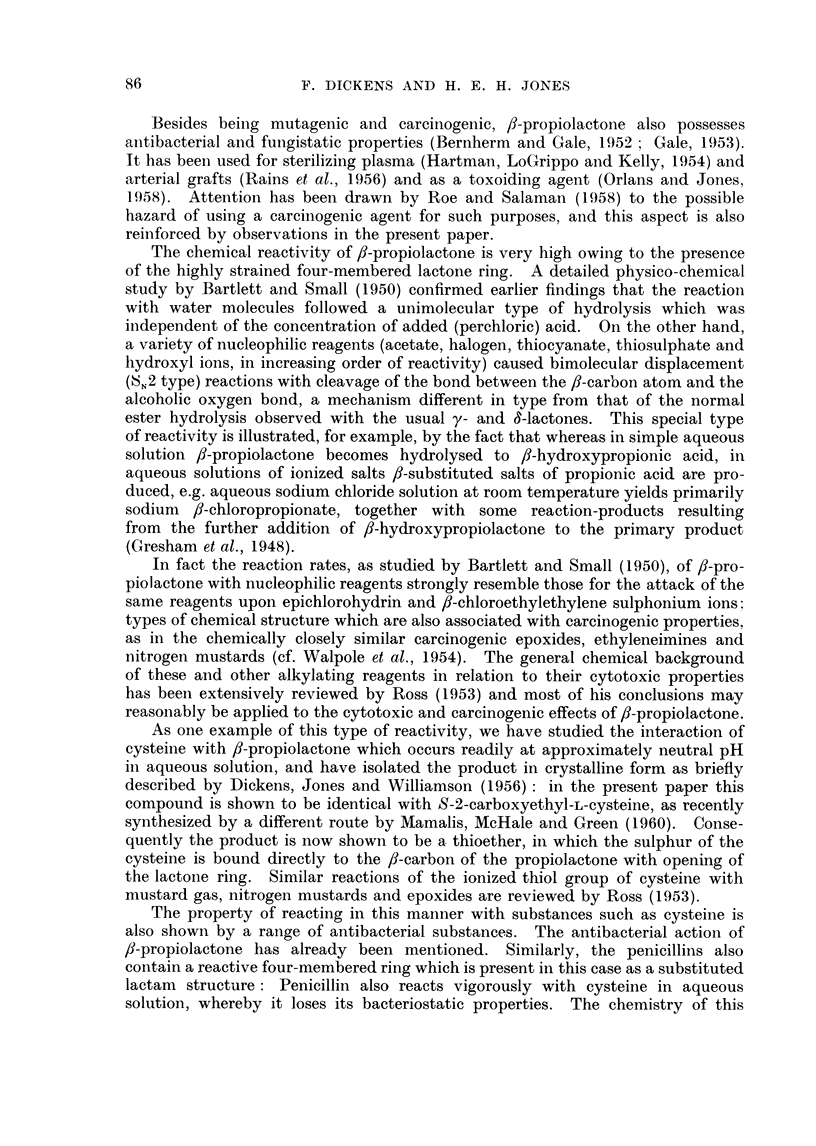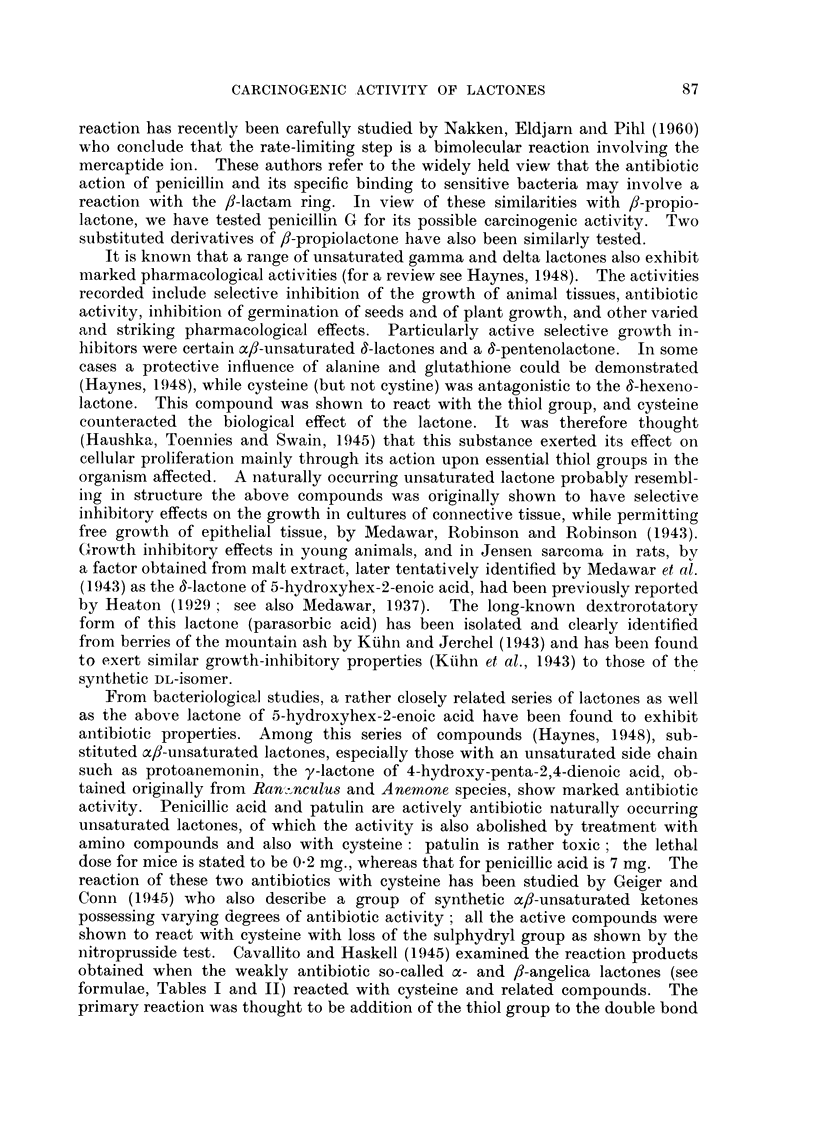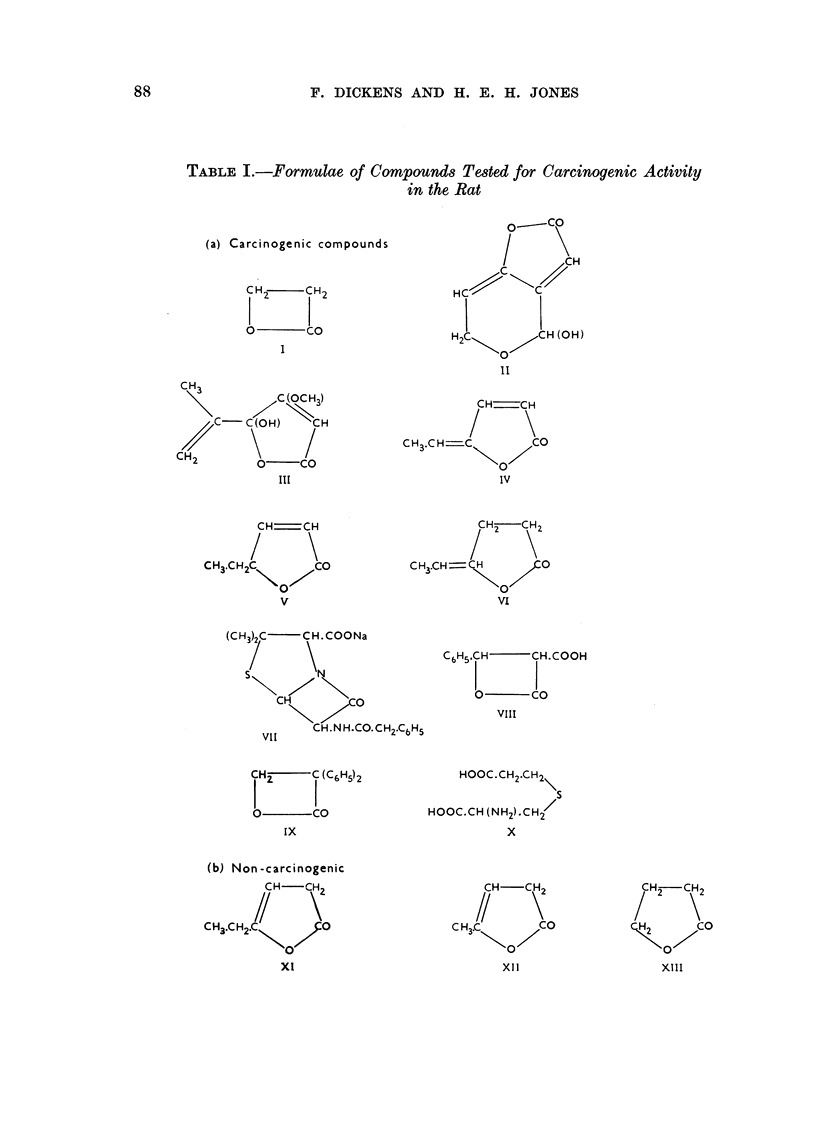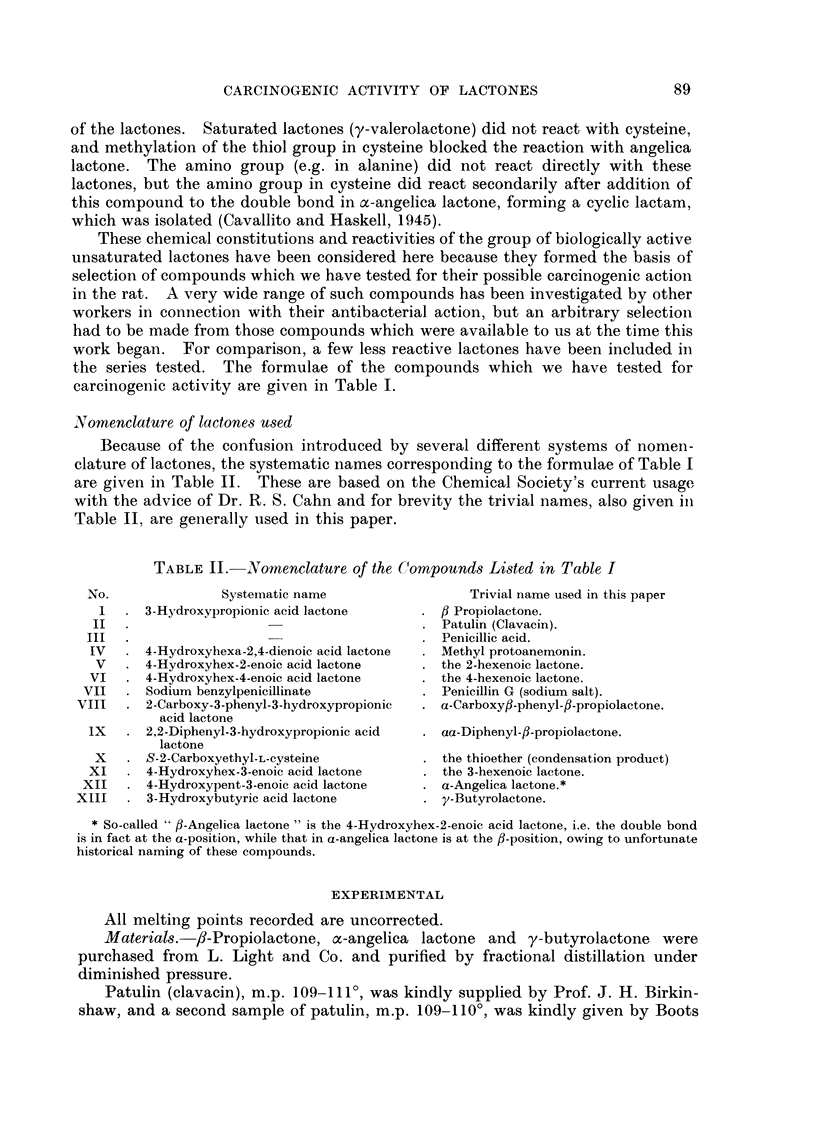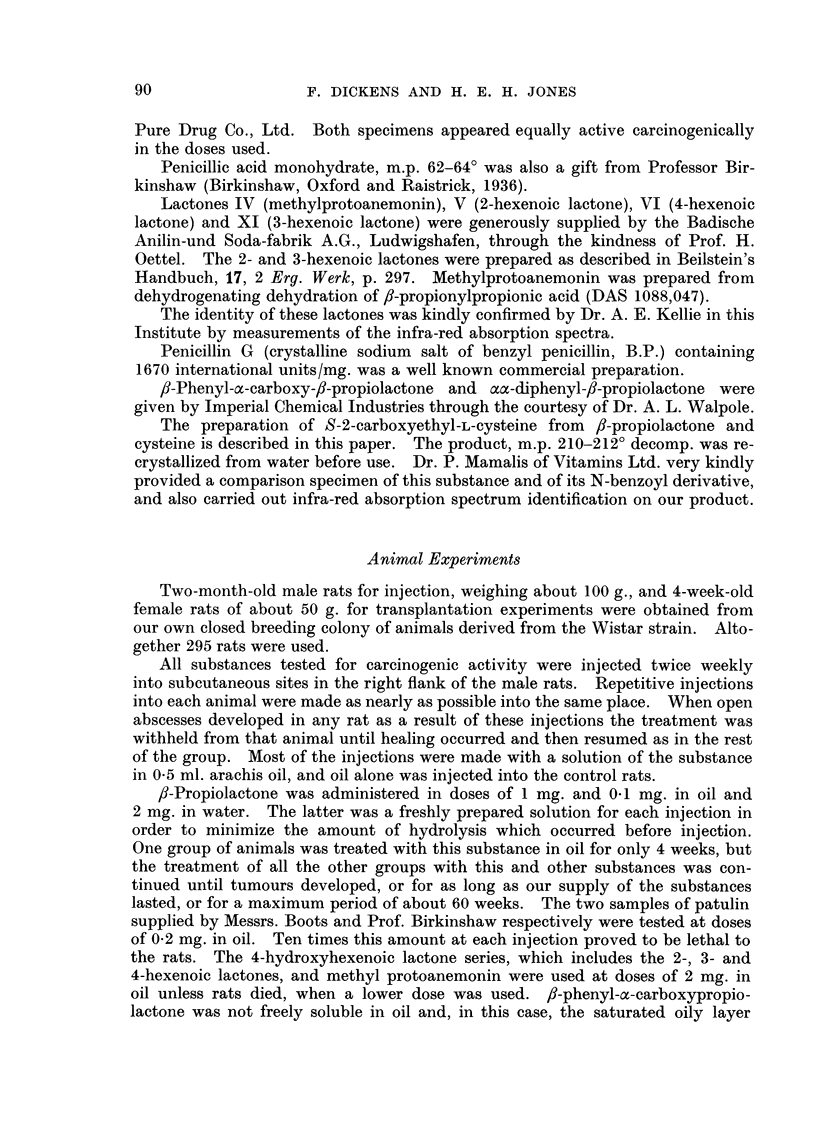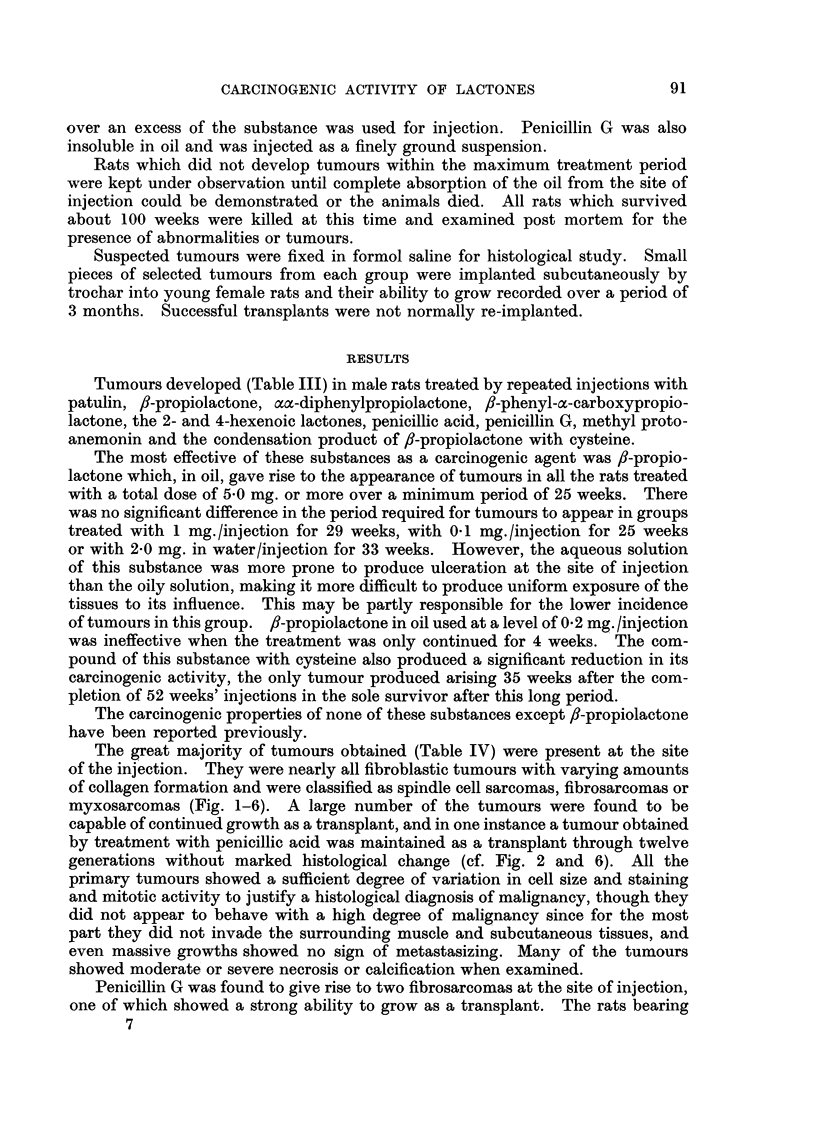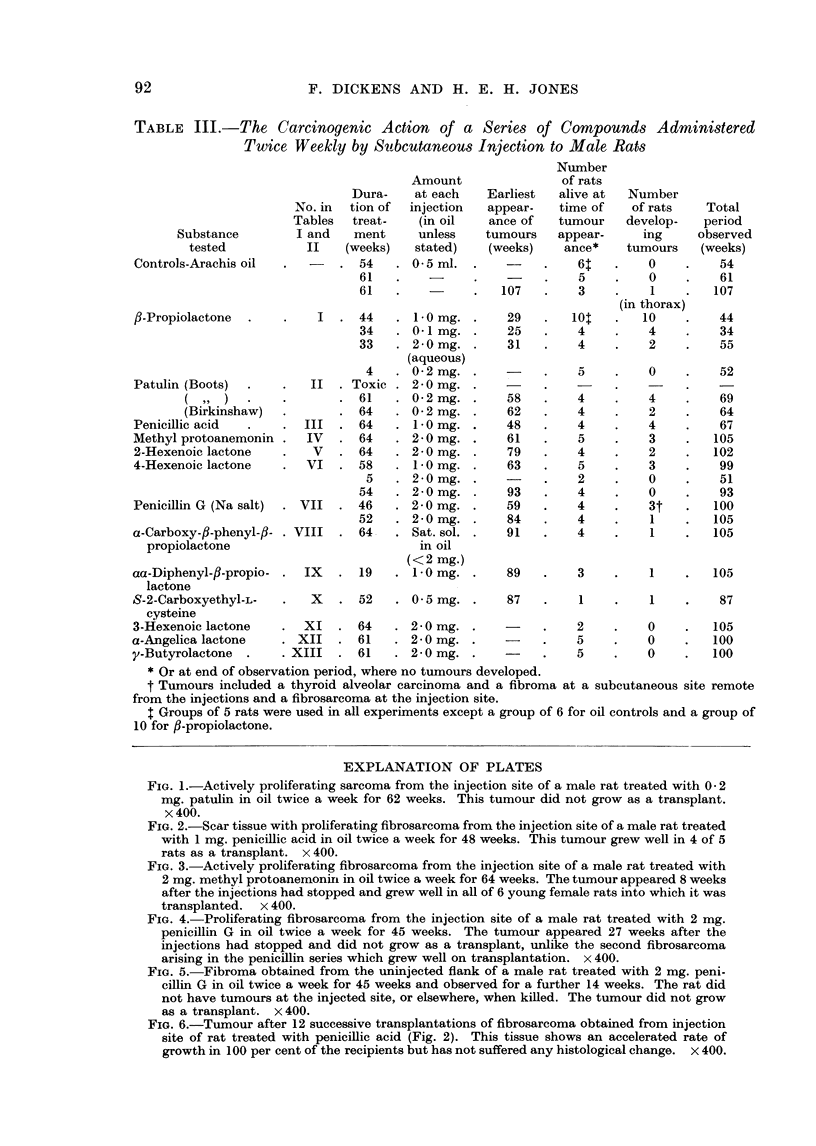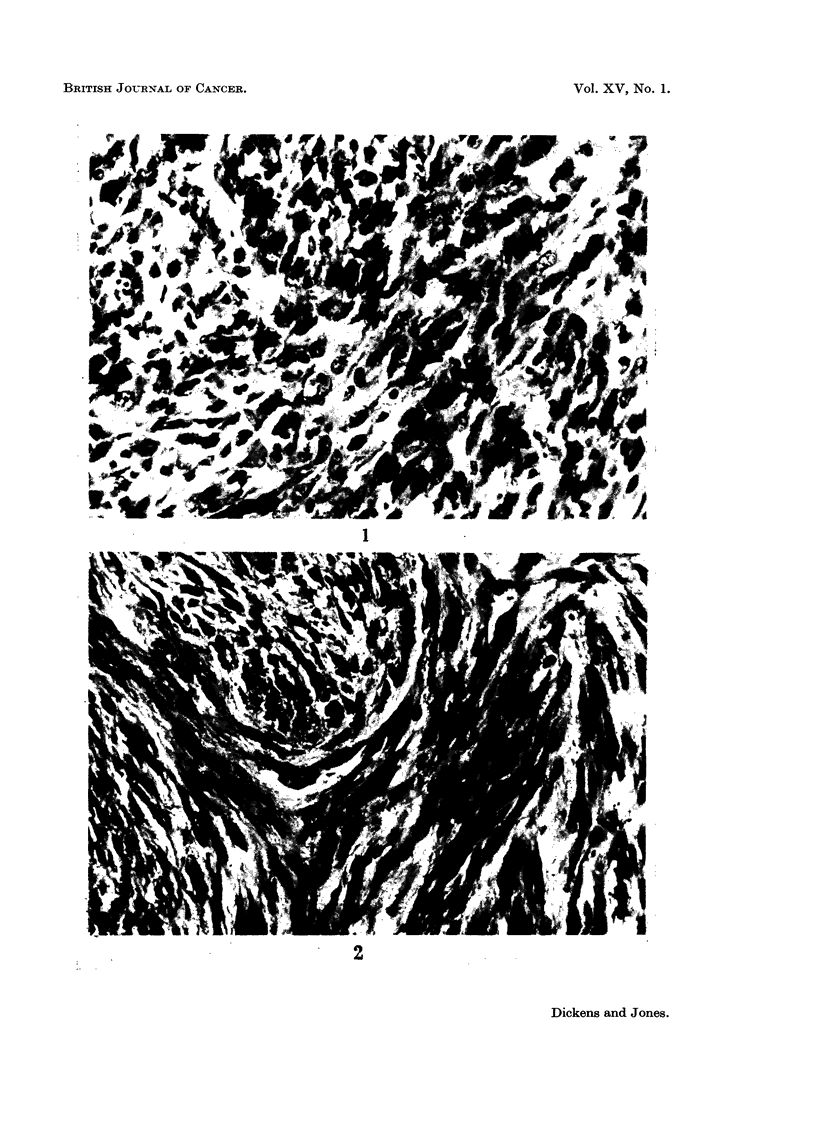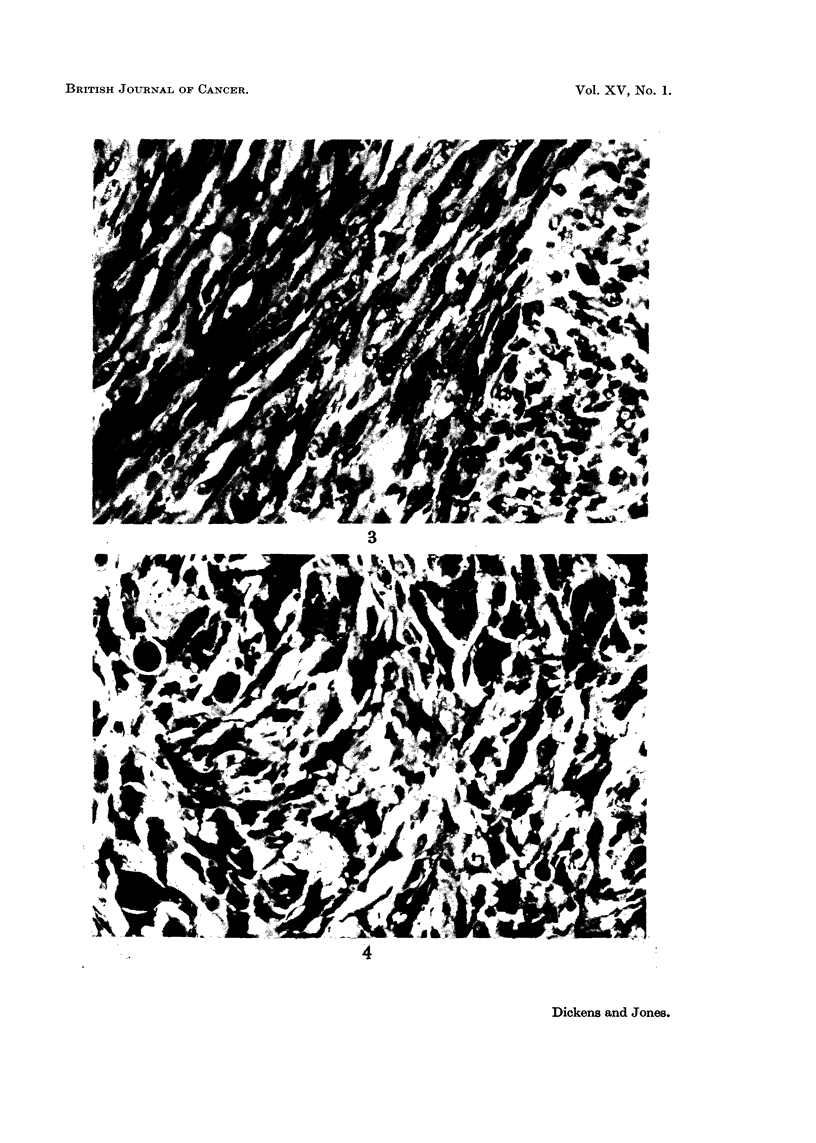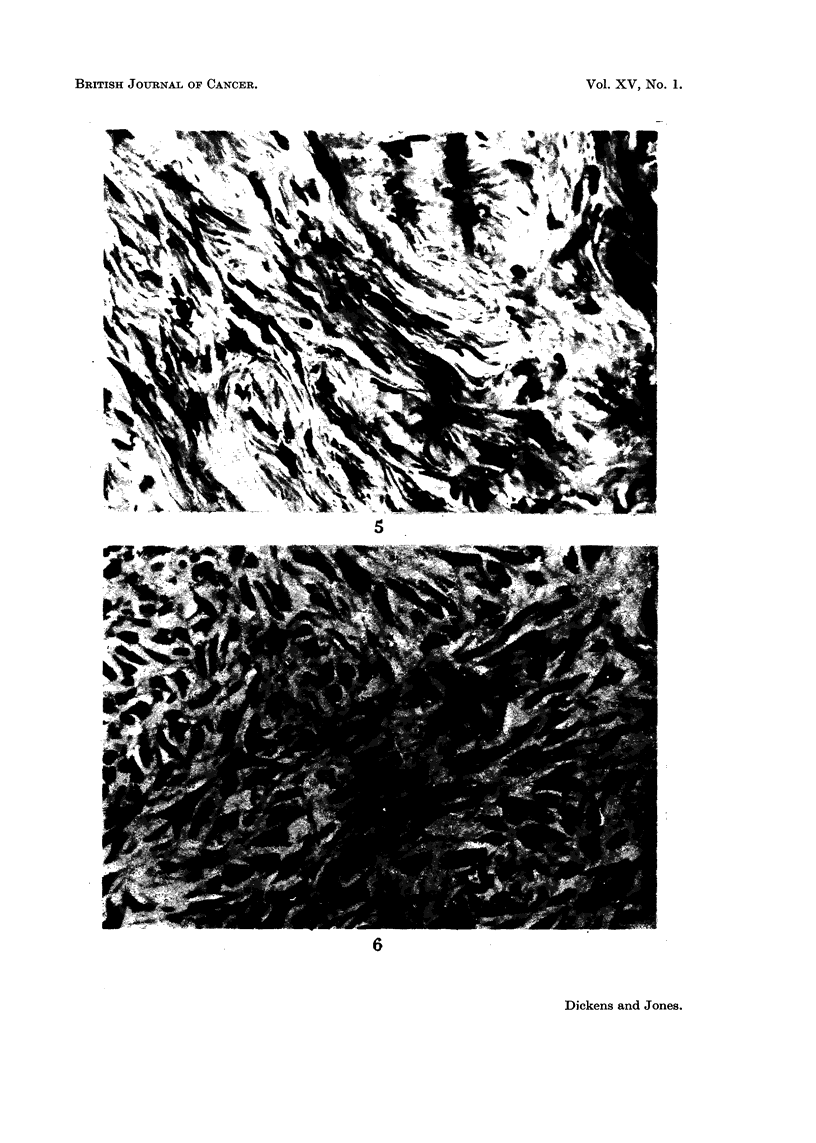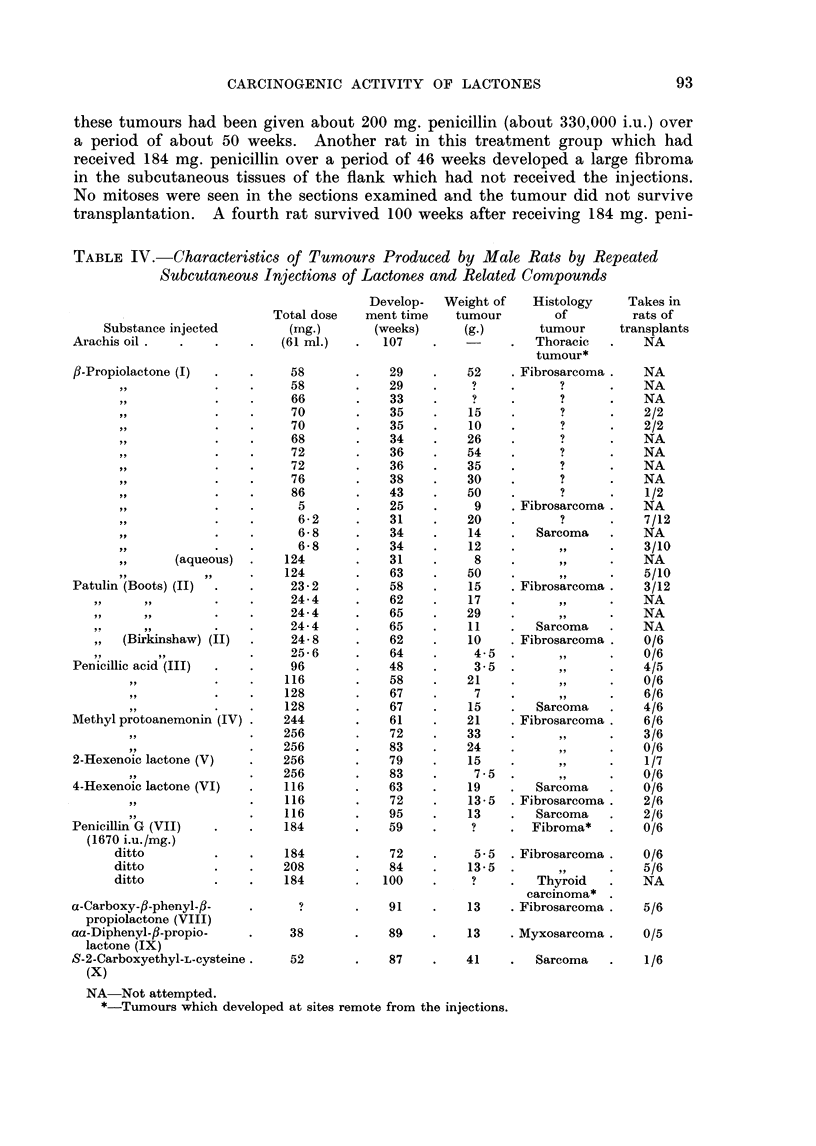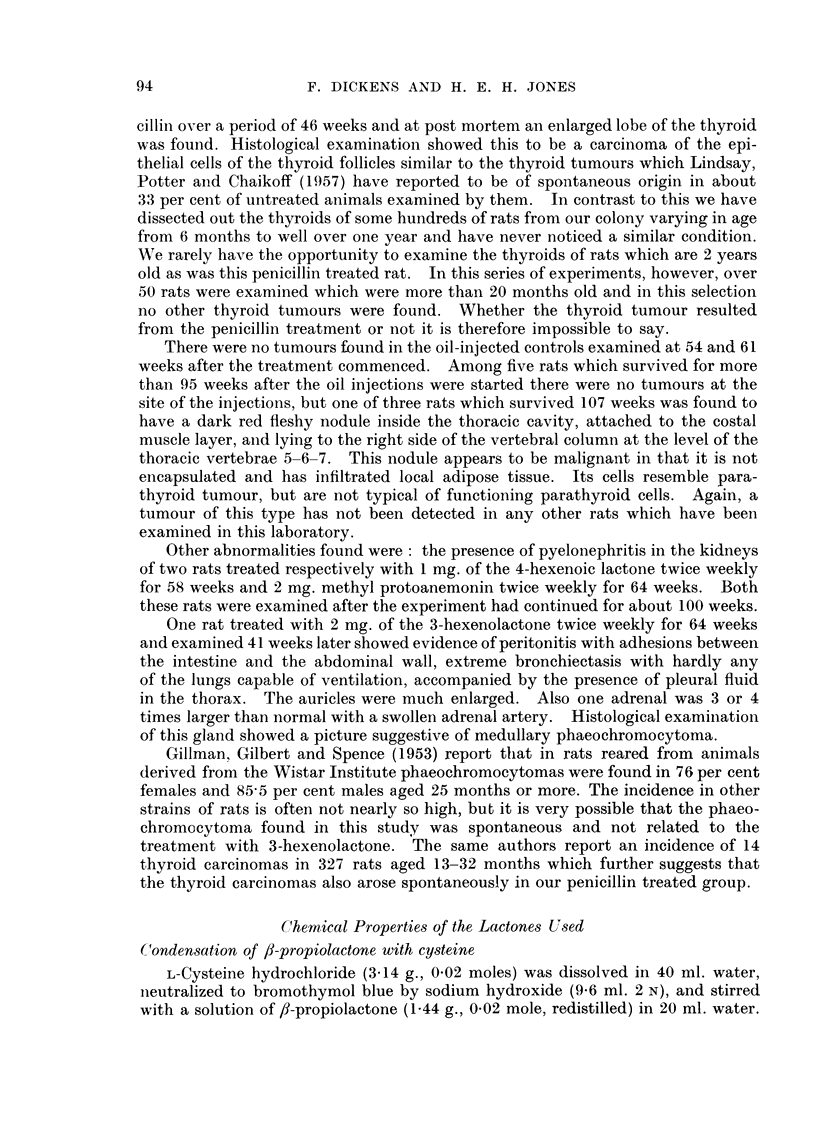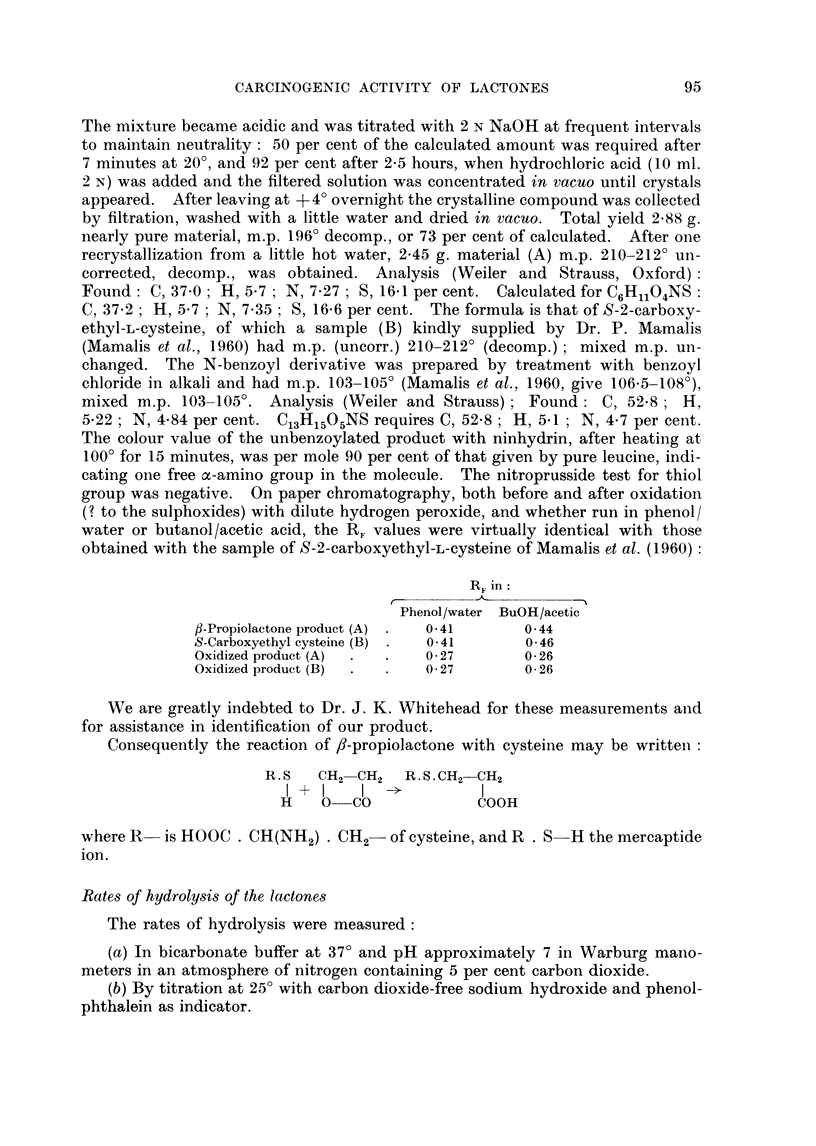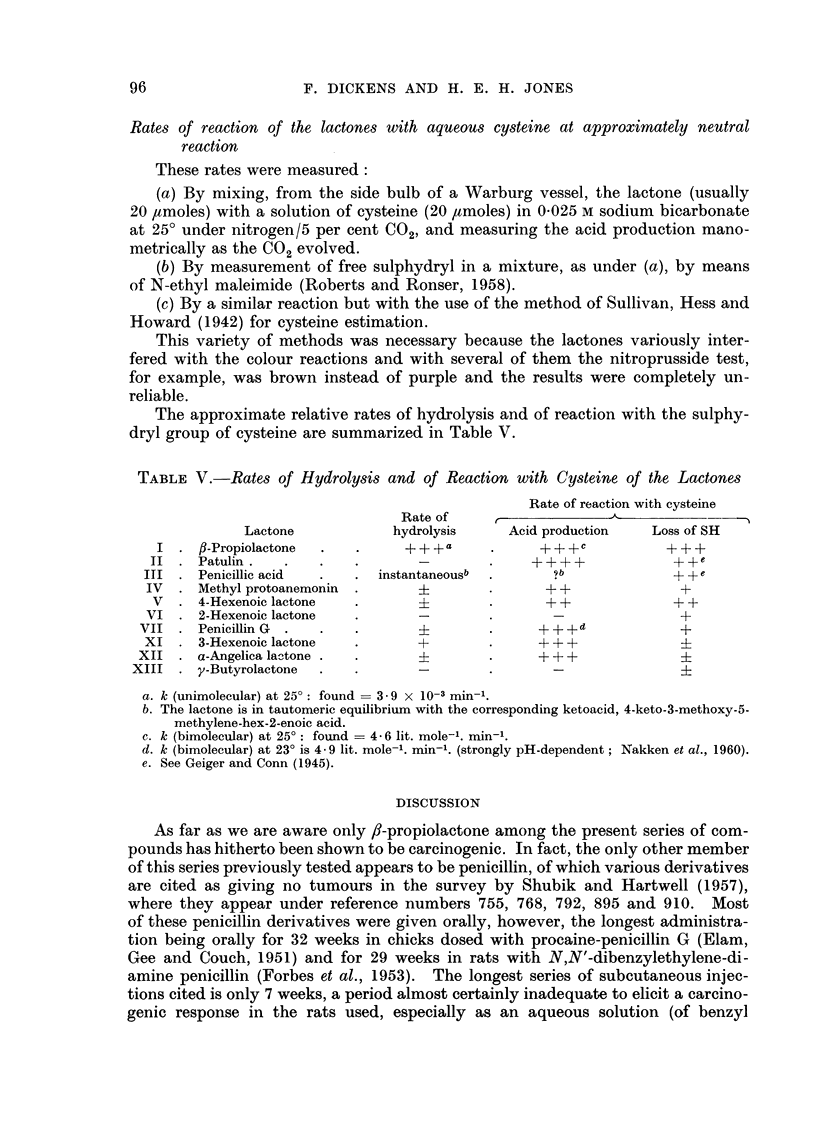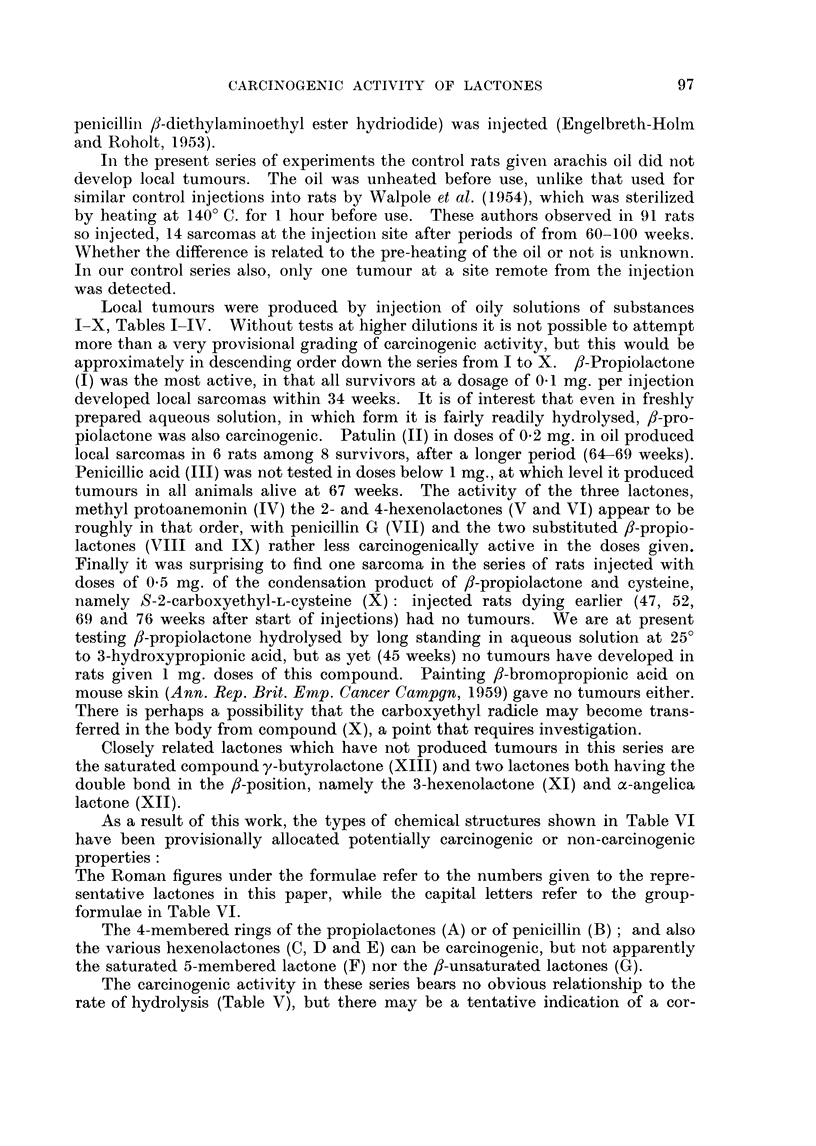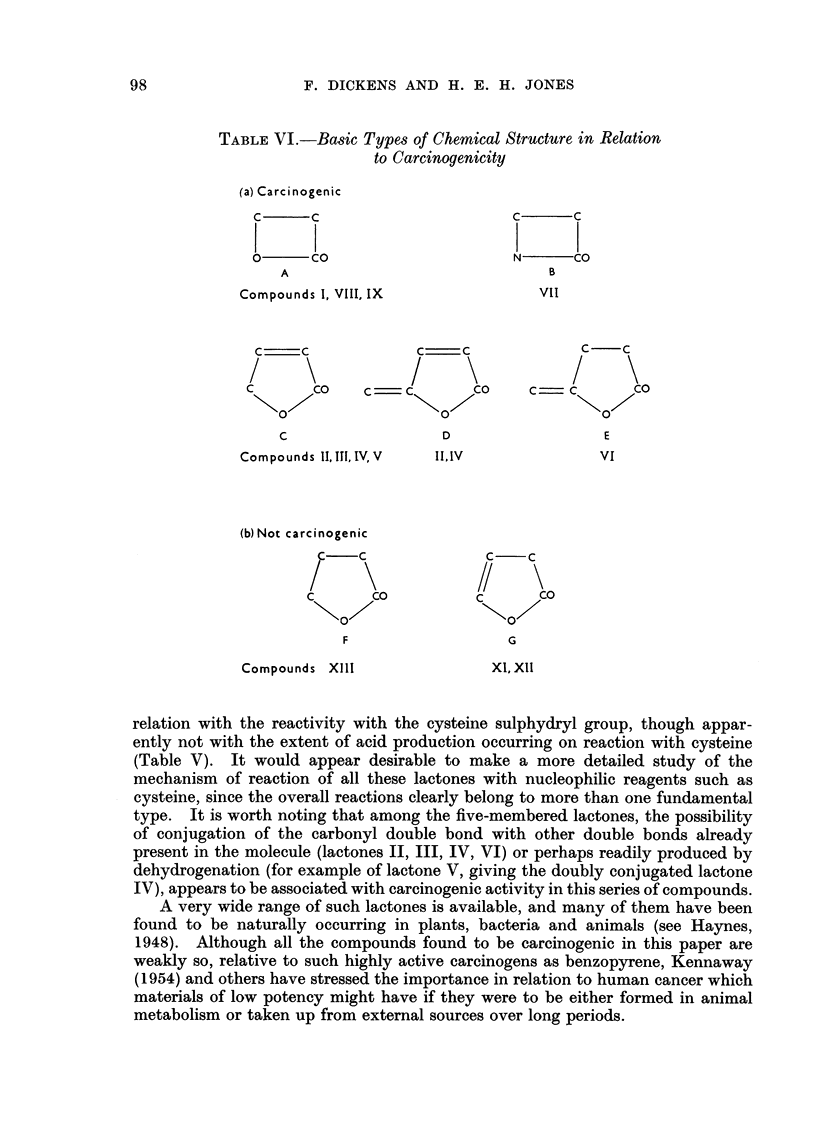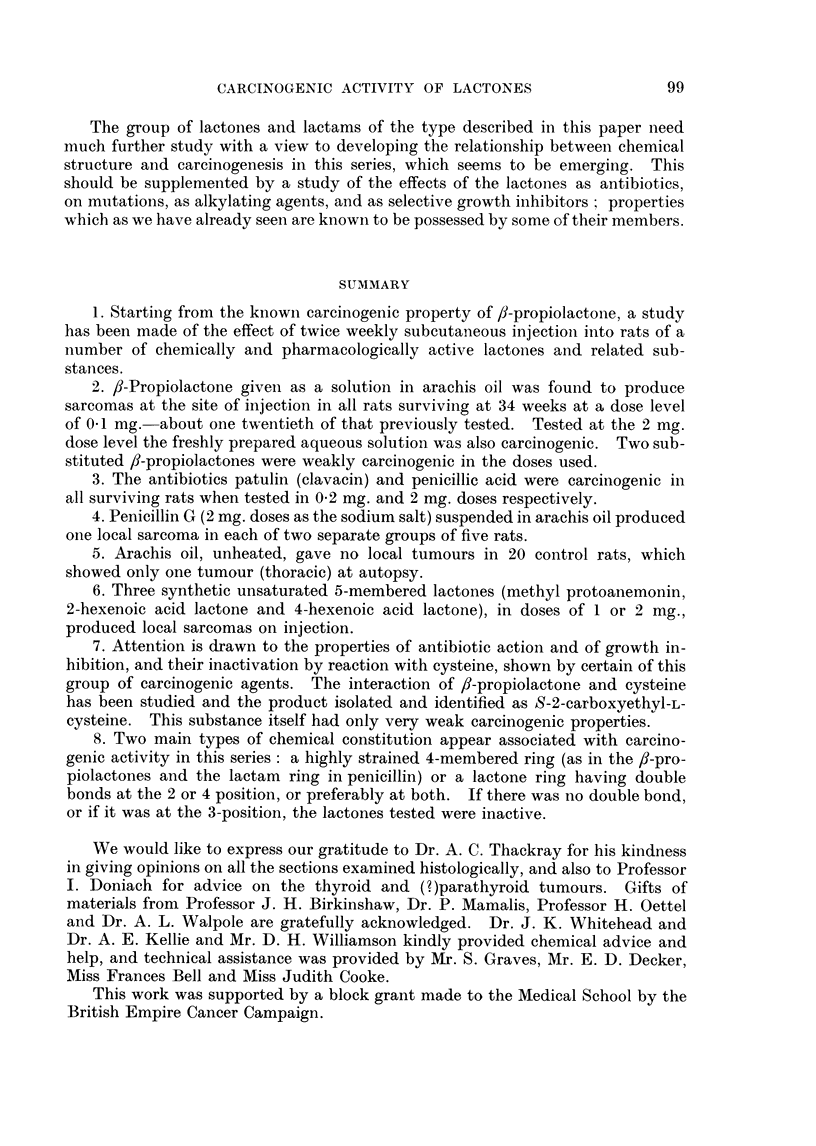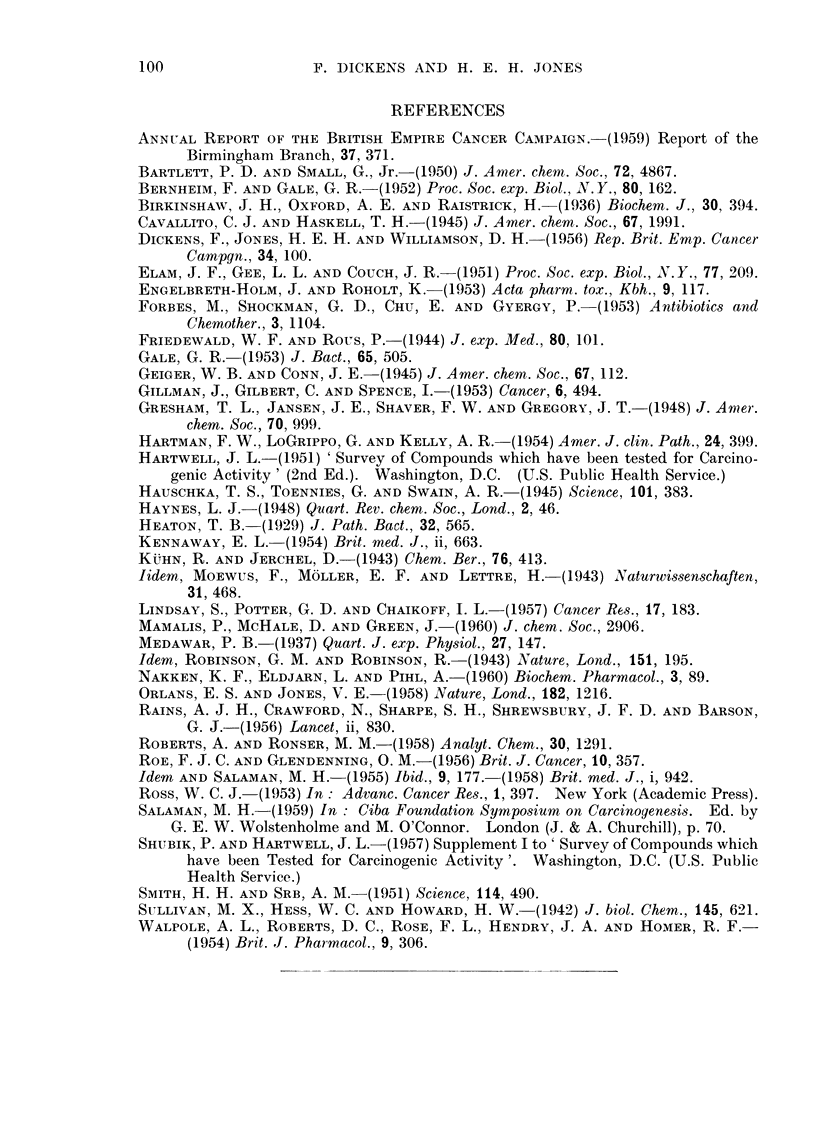# Carcinogenic Activity of a Series of Reactive Lactones and Related Substances

**DOI:** 10.1038/bjc.1961.10

**Published:** 1961-03

**Authors:** F. Dickens, H. E. H. Jones

## Abstract

**Images:**


### CARCINOGENIC ACTIVITY OF A SERIES OF REACTIVE LACTONES AND RELATED SUBSTANCES

F. DICKENS AND H. E. H. JONES

*From the Courtauld Institute of Biochemistry, Middlesex Hospital Medical School, London, W.1*

Received for publication January 31, 1961

ALTHOUGH such a wide variety of chemical substances has been tested for carcinogenic activity, the group of lactones has been largely neglected. Thus, Hartwell (1951) and Shubik and Hartwell (1957) together list 1329 plus 779, a total of 2108, different compounds tested, but very few lactones appear in their comprehensive Survey, and in almost all instances even these have not been tested over any adequate period for their possible carcinogenic action to have been detected.

One of the very few lactones hitherto satisfactorily studied in this respect is $\beta$-propiolactone. This vesicant substance was found to be mutagenic in *Neurospora* by Smith and Srb (1951), a finding which led Walpole *et al.* (1954) to study the effect of prolonged subcutaneous injections extending over 13 weeks into rats of $\beta$-propiolactone, 2 mg./100 g. body weight, dissolved in arachis oil. Of 12 rats so treated, 9 developed sarcomas at the injection site after 28 to 55 weeks from the start of administration. Roe and Salaman (1955) decided that for the skin of the mouse $\beta$-propiolactone was undoubtedly an " initiator " in carcinogenesis in the sense used by Friedewald and Rous (1944) giving tumours rapidly when alternate treatments with croton oil as " promoter " were also given. At first they expressed doubt whether $\beta$-propiolactone is itself carcinogenic for the skin of the mouse, but this was apparently due to too short a period of observation, since Roe and Glendenning (1956) later observed tumours after 27–52 weekly paintings of 2·5 per cent $\beta$-propiolactone in acetone (papillomata arose in 5 mice of 9 treated, becoming malignant in 2 mice after 40 weeks applications). With higher initial doses, the early ulceration and scarring produced were followed by earlier malignant change (3 carcinomas in 20 mice after 21 weeks). Consequently $\beta$-propiolactone must be considered definitely a carcinogenic substance, whether given subcutaneously to rats or by application to the skin in mice. In either case repeated applications are necessary for tumour production ; fewer applications than those given above have failed to produce tumours in mice (Salaman, 1959), and this is also shown in the present paper to apply to subcutaneous injections in the rat. This negative result was in fact to be anticipated from the short period of persistence of the agent, due to the ease of breakdown of this unstable lactone ring in the body (cf. Dickens, Jones and Williamson, 1956). This also applies to a varying extent to the other lactones whose carcinogenic properties are described in this paper, all of which were therefore applied repeatedly over long periods, and usually from an oily depot to assist in slow liberation and elimination.

Besides being mutagenic and carcinogenic, $\beta$-propiolactone also possesses antibacterial and fungistatic properties (Bernherm and Gale, 1952 ; Gale, 1953). It has been used for sterilizing plasma (Hartman, LoGrippo and Kelly, 1954) and arterial grafts (Rains *et al.*, 1956) and as a toxoiding agent (Orlans and Jones, 1958). Attention has been drawn by Roe and Salaman (1958) to the possible hazard of using a carcinogenic agent for such purposes, and this aspect is also reinforced by observations in the present paper.

The chemical reactivity of $\beta$-propiolactone is very high owing to the presence of the highly strained four-membered lactone ring. A detailed physico-chemical study by Bartlett and Small (1950) confirmed earlier findings that the reaction with water molecules followed a unimolecular type of hydrolysis which was independent of the concentration of added (perchloric) acid. On the other hand, a variety of nucleophilic reagents (acetate, halogen, thiocyanate, thiosulphate and hydroxyl ions, in increasing order of reactivity) caused bimolecular displacement ($S_N2$ type) reactions with cleavage of the bond between the $\beta$-carbon atom and the alcoholic oxygen bond, a mechanism different in type from that of the normal ester hydrolysis observed with the usual $\gamma$- and $\delta$-lactones. This special type of reactivity is illustrated, for example, by the fact that whereas in simple aqueous solution $\beta$-propiolactone becomes hydrolysed to $\beta$-hydroxypropionic acid, in aqueous solutions of ionized salts $\beta$-substituted salts of propionic acid are produced, e.g. aqueous sodium chloride solution at room temperature yields primarily sodium $\beta$-chloropropionate, together with some reaction-products resulting from the further addition of $\beta$-hydroxypropiolactone to the primary product (Gresham *et al.*, 1948).

In fact the reaction rates, as studied by Bartlett and Small (1950), of $\beta$-propiolactone with nucleophilic reagents strongly resemble those for the attack of the same reagents upon epichlorohydrin and $\beta$-chloroethylethylene sulphonium ions; types of chemical structure which are also associated with carcinogenic properties, as in the chemically closely similar carcinogenic epoxides, ethyleneimines and nitrogen mustards (cf. Walpole *et al.*, 1954). The general chemical background of these and other alkylating reagents in relation to their cytotoxic properties has been extensively reviewed by Ross (1953) and most of his conclusions may reasonably be applied to the cytotoxic and carcinogenic effects of $\beta$-propiolactone.

As one example of this type of reactivity, we have studied the interaction of cysteine with $\beta$-propiolactone which occurs readily at approximately neutral pH in aqueous solution, and have isolated the product in crystalline form as briefly described by Dickens, Jones and Williamson (1956) : in the present paper this compound is shown to be identical with *S*-2-carboxyethyl-L-cysteine, as recently synthesized by a different route by Mamalis, McHale and Green (1960). Consequently the product is now shown to be a thioether, in which the sulphur of the cysteine is bound directly to the $\beta$-carbon of the propiolactone with opening of the lactone ring. Similar reactions of the ionized thiol group of cysteine with mustard gas, nitrogen mustards and epoxides are reviewed by Ross (1953).

The property of reacting in this manner with substances such as cysteine is also shown by a range of antibacterial substances. The antibacterial action of $\beta$-propiolactone has already been mentioned. Similarly, the penicillins also contain a reactive four-membered ring which is present in this case as a substituted lactam structure : Penicillin also reacts vigorously with cysteine in aqueous solution, whereby it loses its bacteriostatic properties. The chemistry of this reaction has recently been carefully studied by Nakken, Eldjarn and Pihl (1960) who conclude that the rate-limiting step is a bimolecular reaction involving the mercaptide ion. These authors refer to the widely held view that the antibiotic action of penicillin and its specific binding to sensitive bacteria may involve a reaction with the $\beta$-lactam ring. In view of these similarities with $\beta$-propiolactone, we have tested penicillin G for its possible carcinogenic activity. Two substituted derivatives of $\beta$-propiolactone have also been similarly tested.

It is known that a range of unsaturated gamma and delta lactones also exhibit marked pharmacological activities (for a review see Haynes, 1948). The activities recorded include selective inhibition of the growth of animal tissues, antibiotic activity, inhibition of germination of seeds and of plant growth, and other varied and striking pharmacological effects. Particularly active selective growth inhibitors were certain $\alpha\beta$-unsaturated $\delta$-lactones and a $\delta$-pentenolactone. In some cases a protective influence of alanine and glutathione could be demonstrated (Haynes, 1948), while cysteine (but not cystine) was antagonistic to the $\delta$-hexenolactone. This compound was shown to react with the thiol group, and cysteine counteracted the biological effect of the lactone. It was therefore thought (Haushka, Toennies and Swain, 1945) that this substance exerted its effect on cellular proliferation mainly through its action upon essential thiol groups in the organism affected. A naturally occurring unsaturated lactone probably resembling in structure the above compounds was originally shown to have selective inhibitory effects on the growth in cultures of connective tissue, while permitting free growth of epithelial tissue, by Medawar, Robinson and Robinson (1943). Growth inhibitory effects in young animals, and in Jensen sarcoma in rats, by a factor obtained from malt extract, later tentatively identified by Medawar *et al.* (1943) as the $\delta$-lactone of 5-hydroxyhex-2-enoic acid, had been previously reported by Heaton (1929 ; see also Medawar, 1937). The long-known dextrorotatory form of this lactone (parasorbic acid) has been isolated and clearly identified from berries of the mountain ash by Kühn and Jerchel (1943) and has been found to exert similar growth-inhibitory properties (Kühn *et al.*, 1943) to those of the synthetic DL-isomer.

From bacteriological studies, a rather closely related series of lactones as well as the above lactone of 5-hydroxyhex-2-enoic acid have been found to exhibit antibiotic properties. Among this series of compounds (Haynes, 1948), substituted $\alpha\beta$-unsaturated lactones, especially those with an unsaturated side chain such as protoanemonin, the $\gamma$-lactone of 4-hydroxy-penta-2,4-dienoic acid, obtained originally from *Ranunculus* and *Anemone* species, show marked antibiotic activity. Penicillic acid and patulin are actively antibiotic naturally occurring unsaturated lactones, of which the activity is also abolished by treatment with amino compounds and also with cysteine : patulin is rather toxic ; the lethal dose for mice is stated to be 0·2 mg., whereas that for penicillic acid is 7 mg. The reaction of these two antibiotics with cysteine has been studied by Geiger and Conn (1945) who also describe a group of synthetic $\alpha\beta$-unsaturated ketones possessing varying degrees of antibiotic activity ; all the active compounds were shown to react with cysteine with loss of the sulphydryl group as shown by the nitroprusside test. Cavallito and Haskell (1945) examined the reaction products obtained when the weakly antibiotic so-called $\alpha$- and $\beta$-angelica lactones (see formulae, Tables I and II) reacted with cysteine and related compounds. The primary reaction was thought to be addition of the thiol group to the double bond

**Table I.**—*Formulae of Compounds Tested for Carcinogenic Activity in the Rat*

(a) Carcinogenic compounds

I

II

III

IV

V

VI

VII

VIII

IX

X

(b) Non-carcinogenic

XI

XII

XIII

of the lactones. Saturated lactones ($\gamma$-valerolactone) did not react with cysteine, and methylation of the thiol group in cysteine blocked the reaction with angelica lactone. The amino group (e.g. in alanine) did not react directly with these lactones, but the amino group in cysteine did react secondarily after addition of this compound to the double bond in $\alpha$-angelica lactone, forming a cyclic lactam, which was isolated (Cavallito and Haskell, 1945).

These chemical constitutions and reactivities of the group of biologically active unsaturated lactones have been considered here because they formed the basis of selection of compounds which we have tested for their possible carcinogenic action in the rat. A very wide range of such compounds has been investigated by other workers in connection with their antibacterial action, but an arbitrary selection had to be made from those compounds which were available to us at the time this work began. For comparison, a few less reactive lactones have been included in the series tested. The formulae of the compounds which we have tested for carcinogenic activity are given in Table I.

*Nomenclature of lactones used*

Because of the confusion introduced by several different systems of nomenclature of lactones, the systematic names corresponding to the formulae of Table I are given in Table II. These are based on the Chemical Society's current usage with the advice of Dr. R. S. Cahn and for brevity the trivial names, also given in Table II, are generally used in this paper.

TABLE II.—*Nomenclature of the Compounds Listed in Table I*

| No. | Systematic name | Trivial name used in this paper |
|---|---|---|
| I | 3-Hydroxypropionic acid lactone | $\beta$ Propiolactone. |
| II | — | Patulin (Clavacin). |
| III | — | Penicillic acid. |
| IV | 4-Hydroxyhexa-2,4-dienoic acid lactone | Methyl protoanemonin. |
| V | 4-Hydroxyhex-2-enoic acid lactone | the 2-hexenoic lactone. |
| VI | 4-Hydroxyhex-4-enoic acid lactone | the 4-hexenoic lactone. |
| VII | Sodium benzylpenicillinate | Penicillin G (sodium salt). |
| VIII | 2-Carboxy-3-phenyl-3-hydroxypropionic acid lactone | $\alpha$-Carboxy$\beta$-phenyl-$\beta$-propiolactone. |
| IX | 2,2-Diphenyl-3-hydroxypropionic acid lactone | $\alpha\alpha$-Diphenyl-$\beta$-propiolactone. |
| X | *S*-2-Carboxyethyl-L-cysteine | the thioether (condensation product) |
| XI | 4-Hydroxyhex-3-enoic acid lactone | the 3-hexenoic lactone. |
| XII | 4-Hydroxypent-3-enoic acid lactone | $\alpha$-Angelica lactone.* |
| XIII | 3-Hydroxybutyric acid lactone | $\gamma$-Butyrolactone. |

* So-called "$\beta$-Angelica lactone" is the 4-Hydroxyhex-2-enoic acid lactone, i.e. the double bond is in fact at the $\alpha$-position, while that in $\alpha$-angelica lactone is at the $\beta$-position, owing to unfortunate historical naming of these compounds.

### EXPERIMENTAL

All melting points recorded are uncorrected.

*Materials.*—$\beta$-Propiolactone, $\alpha$-angelica lactone and $\gamma$-butyrolactone were purchased from L. Light and Co. and purified by fractional distillation under diminished pressure.

Patulin (clavacin), m.p. 109–111°, was kindly supplied by Prof. J. H. Birkinshaw, and a second sample of patulin, m.p. 109–110°, was kindly given by Boots

Pure Drug Co., Ltd. Both specimens appeared equally active carcinogenically in the doses used.

Penicillic acid monohydrate, m.p. 62–64° was also a gift from Professor Birkinshaw (Birkinshaw, Oxford and Raistrick, 1936).

Lactones IV (methylprotoanemonin), V (2-hexenoic lactone), VI (4-hexenoic lactone) and XI (3-hexenoic lactone) were generously supplied by the Badische Anilin-und Soda-fabrik A.G., Ludwigshafen, through the kindness of Prof. H. Oettel. The 2- and 3-hexenoic lactones were prepared as described in Beilstein's Handbuch, **17**, 2 *Erg. Werk*, p. 297. Methylprotoanemonin was prepared from dehydrogenating dehydration of $\beta$-propionylpropionic acid (DAS 1088,047).

The identity of these lactones was kindly confirmed by Dr. A. E. Kellie in this Institute by measurements of the infra-red absorption spectra.

Penicillin G (crystalline sodium salt of benzyl penicillin, B.P.) containing 1670 international units/mg. was a well known commercial preparation.

$\beta$-Phenyl-$\alpha$-carboxy-$\beta$-propiolactone and $\alpha\alpha$-diphenyl-$\beta$-propiolactone were given by Imperial Chemical Industries through the courtesy of Dr. A. L. Walpole.

The preparation of *S*-2-carboxyethyl-L-cysteine from $\beta$-propiolactone and cysteine is described in this paper. The product, m.p. 210–212° decomp. was recrystallized from water before use. Dr. P. Mamalis of Vitamins Ltd. very kindly provided a comparison specimen of this substance and of its N-benzoyl derivative, and also carried out infra-red absorption spectrum identification on our product.

### *Animal Experiments*

Two-month-old male rats for injection, weighing about 100 g., and 4-week-old female rats of about 50 g. for transplantation experiments were obtained from our own closed breeding colony of animals derived from the Wistar strain. Altogether 295 rats were used.

All substances tested for carcinogenic activity were injected twice weekly into subcutaneous sites in the right flank of the male rats. Repetitive injections into each animal were made as nearly as possible into the same place. When open abscesses developed in any rat as a result of these injections the treatment was withheld from that animal until healing occurred and then resumed as in the rest of the group. Most of the injections were made with a solution of the substance in 0·5 ml. arachis oil, and oil alone was injected into the control rats.

$\beta$-Propiolactone was administered in doses of 1 mg. and 0·1 mg. in oil and 2 mg. in water. The latter was a freshly prepared solution for each injection in order to minimize the amount of hydrolysis which occurred before injection. One group of animals was treated with this substance in oil for only 4 weeks, but the treatment of all the other groups with this and other substances was continued until tumours developed, or for as long as our supply of the substances lasted, or for a maximum period of about 60 weeks. The two samples of patulin supplied by Messrs. Boots and Prof. Birkinshaw respectively were tested at doses of 0·2 mg. in oil. Ten times this amount at each injection proved to be lethal to the rats. The 4-hydroxyhexenoic lactone series, which includes the 2-, 3- and 4-hexenoic lactones, and methyl protoanemonin were used at doses of 2 mg. in oil unless rats died, when a lower dose was used. $\beta$-phenyl-$\alpha$-carboxypropiolactone was not freely soluble in oil and, in this case, the saturated oily layer over an excess of the substance was used for injection. Penicillin G was also insoluble in oil and was injected as a finely ground suspension.

Rats which did not develop tumours within the maximum treatment period were kept under observation until complete absorption of the oil from the site of injection could be demonstrated or the animals died. All rats which survived about 100 weeks were killed at this time and examined post mortem for the presence of abnormalities or tumours.

Suspected tumours were fixed in formol saline for histological study. Small pieces of selected tumours from each group were implanted subcutaneously by trochar into young female rats and their ability to grow recorded over a period of 3 months. Successful transplants were not normally re-implanted.

### RESULTS

Tumours developed (Table III) in male rats treated by repeated injections with patulin, $\beta$-propiolactone, $\alpha\alpha$-diphenylpropiolactone, $\beta$-phenyl-$\alpha$-carboxypropiolactone, the 2- and 4-hexenoic lactones, penicillic acid, penicillin G, methyl protoanemonin and the condensation product of $\beta$-propiolactone with cysteine.

The most effective of these substances as a carcinogenic agent was $\beta$-propiolactone which, in oil, gave rise to the appearance of tumours in all the rats treated with a total dose of 5·0 mg. or more over a minimum period of 25 weeks. There was no significant difference in the period required for tumours to appear in groups treated with 1 mg./injection for 29 weeks, with 0·1 mg./injection for 25 weeks or with 2·0 mg. in water/injection for 33 weeks. However, the aqueous solution of this substance was more prone to produce ulceration at the site of injection than the oily solution, making it more difficult to produce uniform exposure of the tissues to its influence. This may be partly responsible for the lower incidence of tumours in this group. $\beta$-propiolactone in oil used at a level of 0·2 mg./injection was ineffective when the treatment was only continued for 4 weeks. The compound of this substance with cysteine also produced a significant reduction in its carcinogenic activity, the only tumour produced arising 35 weeks after the completion of 52 weeks' injections in the sole survivor after this long period.

The carcinogenic properties of none of these substances except $\beta$-propiolactone have been reported previously.

The great majority of tumours obtained (Table IV) were present at the site of the injection. They were nearly all fibroblastic tumours with varying amounts of collagen formation and were classified as spindle cell sarcomas, fibrosarcomas or myxosarcomas (Fig. 1–6). A large number of the tumours were found to be capable of continued growth as a transplant, and in one instance a tumour obtained by treatment with penicillic acid was maintained as a transplant through twelve generations without marked histological change (cf. Fig. 2 and 6). All the primary tumours showed a sufficient degree of variation in cell size and staining and mitotic activity to justify a histological diagnosis of malignancy, though they did not appear to behave with a high degree of malignancy since for the most part they did not invade the surrounding muscle and subcutaneous tissues, and even massive growths showed no sign of metastasizing. Many of the tumours showed moderate or severe necrosis or calcification when examined.

Penicillin G was found to give rise to two fibrosarcomas at the site of injection, one of which showed a strong ability to grow as a transplant. The rats bearing

TABLE III.—*The Carcinogenic Action of a Series of Compounds Administered Twice Weekly by Subcutaneous Injection to Male Rats*

| Substance tested | No. in Tables I and II | Duration of treatment (weeks) | Amount at each injection (in oil unless stated) | Earliest appearance of tumours (weeks) | Number of rats alive at time of tumour appearance* | Number of rats developing tumours | Total period observed (weeks) |
|---|---|---|---|---|---|---|---|
| Controls-Arachis oil | — | 54 | 0·5 ml. | — | 6‡ | 0 | 54 |
| | | 61 | — | — | 5 | 0 | 61 |
| | | 61 | — | 107 | 3 | 1 (in thorax) | 107 |
| β-Propiolactone | I | 44 | 1·0 mg. | 29 | 10‡ | 10 | 44 |
| | | 34 | 0·1 mg. | 25 | 4 | 4 | 34 |
| | | 33 | 2·0 mg. (aqueous) | 31 | 4 | 2 | 55 |
| | | 4 | 0·2 mg. | — | 5 | 0 | 52 |
| Patulin (Boots) | II | Toxic | 2·0 mg. | — | — | — | — |
| ( „ ) | | 61 | 0·2 mg. | 58 | 4 | 4 | 69 |
| (Birkinshaw) | | 64 | 0·2 mg. | 62 | 4 | 2 | 64 |
| Penicillic acid | III | 64 | 1·0 mg. | 48 | 4 | 4 | 67 |
| Methyl protoanemonin | IV | 64 | 2·0 mg. | 61 | 5 | 3 | 105 |
| 2-Hexenoic lactone | V | 64 | 2·0 mg. | 79 | 4 | 2 | 102 |
| 4-Hexenoic lactone | VI | 58 | 1·0 mg. | 63 | 5 | 3 | 99 |
| | | 5 | 2·0 mg. | — | 2 | 0 | 51 |
| | | 54 | 2·0 mg. | 93 | 4 | 0 | 93 |
| Penicillin G (Na salt) | VII | 46 | 2·0 mg. | 59 | 4 | 3† | 100 |
| | | 52 | 2·0 mg. | 84 | 4 | 1 | 105 |
| α-Carboxy-β-phenyl-β-propiolactone | VIII | 64 | Sat. sol. in oil (<2 mg.) | 91 | 4 | 1 | 105 |
| αα-Diphenyl-β-propiolactone | IX | 19 | 1·0 mg. | 89 | 3 | 1 | 105 |
| *S*-2-Carboxyethyl-L-cysteine | X | 52 | 0·5 mg. | 87 | 1 | 1 | 87 |
| 3-Hexenoic lactone | XI | 64 | 2·0 mg. | — | 2 | 0 | 105 |
| α-Angelica lactone | XII | 61 | 2·0 mg. | — | 5 | 0 | 100 |
| γ-Butyrolactone | XIII | 61 | 2·0 mg. | — | 5 | 0 | 100 |

* Or at end of observation period, where no tumours developed.

† Tumours included a thyroid alveolar carcinoma and a fibroma at a subcutaneous site remote from the injections and a fibrosarcoma at the injection site.

‡ Groups of 5 rats were used in all experiments except a group of 6 for oil controls and a group of 10 for β-propiolactone.

---

### EXPLANATION OF PLATES

FIG. 1.—Actively proliferating sarcoma from the injection site of a male rat treated with 0·2 mg. patulin in oil twice a week for 62 weeks. This tumour did not grow as a transplant. ×400.

FIG. 2.—Scar tissue with proliferating fibrosarcoma from the injection site of a male rat treated with 1 mg. penicillic acid in oil twice a week for 48 weeks. This tumour grew well in 4 of 5 rats as a transplant. ×400.

FIG. 3.—Actively proliferating fibrosarcoma from the injection site of a male rat treated with 2 mg. methyl protoanemonin in oil twice a week for 64 weeks. The tumour appeared 8 weeks after the injections had stopped and grew well in all of 6 young female rats into which it was transplanted. ×400.

FIG. 4.—Proliferating fibrosarcoma from the injection site of a male rat treated with 2 mg. penicillin G in oil twice a week for 45 weeks. The tumour appeared 27 weeks after the injections had stopped and did not grow as a transplant, unlike the second fibrosarcoma arising in the penicillin series which grew well on transplantation. ×400.

FIG. 5.—Fibroma obtained from the uninjected flank of a male rat treated with 2 mg. penicillin G in oil twice a week for 45 weeks and observed for a further 14 weeks. The rat did not have tumours at the injected site, or elsewhere, when killed. The tumour did not grow as a transplant. ×400.

FIG. 6.—Tumour after 12 successive transplantations of fibrosarcoma obtained from injection site of rat treated with penicillic acid (Fig. 2). This tissue shows an accelerated rate of growth in 100 per cent of the recipients but has not suffered any histological change. ×400.

1

2

3

4

Dickens and Jones.

5

6

Dickens and Jones.

these tumours had been given about 200 mg. penicillin (about 330,000 i.u.) over a period of about 50 weeks. Another rat in this treatment group which had received 184 mg. penicillin over a period of 46 weeks developed a large fibroma in the subcutaneous tissues of the flank which had not received the injections. No mitoses were seen in the sections examined and the tumour did not survive transplantation. A fourth rat survived 100 weeks after receiving 184 mg. peni-

TABLE IV.—*Characteristics of Tumours Produced by Male Rats by Repeated Subcutaneous Injections of Lactones and Related Compounds*

| Substance injected | Total dose (mg.) | Development time (weeks) | Weight of tumour (g.) | Histology of tumour | Takes in rats of transplants |
|---|---|---|---|---|---|
| Arachis oil | (61 ml.) | 107 | — | Thoracic tumour* | NA |
| β-Propiolactone (I) | 58 | 29 | 52 | Fibrosarcoma | NA |
| ,, | 58 | 29 | ? | ? | NA |
| ,, | 66 | 33 | ? | ? | NA |
| ,, | 70 | 35 | 15 | ? | 2/2 |
| ,, | 70 | 35 | 10 | ? | 2/2 |
| ,, | 68 | 34 | 26 | ? | NA |
| ,, | 72 | 36 | 54 | ? | NA |
| ,, | 72 | 36 | 35 | ? | NA |
| ,, | 76 | 38 | 30 | ? | NA |
| ,, | 86 | 43 | 50 | ? | 1/2 |
| ,, | 5 | 25 | 9 | Fibrosarcoma | NA |
| ,, | 6·2 | 31 | 20 | ? | 7/12 |
| ,, | 6·8 | 34 | 14 | Sarcoma | NA |
| ,, | 6·8 | 34 | 12 | ,, | 3/10 |
| ,, (aqueous) | 124 | 31 | 8 | ,, | NA |
| ,, ,, | 124 | 63 | 50 | ,, | 5/10 |
| Patulin (Boots) (II) | 23·2 | 58 | 15 | Fibrosarcoma | 3/12 |
| ,, ,, | 24·4 | 62 | 17 | ,, | NA |
| ,, ,, | 24·4 | 65 | 29 | ,, | NA |
| ,, ,, | 24·4 | 65 | 11 | Sarcoma | NA |
| ,, (Birkinshaw) (II) | 24·8 | 62 | 10 | Fibrosarcoma | 0/6 |
| ,, ,, | 25·6 | 64 | 4·5 | ,, | 0/6 |
| Penicillic acid (III) | 96 | 48 | 3·5 | ,, | 4/5 |
| ,, | 116 | 58 | 21 | ,, | 0/6 |
| ,, | 128 | 67 | 7 | ,, | 6/6 |
| ,, | 128 | 67 | 15 | Sarcoma | 4/6 |
| Methyl protoanemonin (IV) | 244 | 61 | 21 | Fibrosarcoma | 6/6 |
| ,, | 256 | 72 | 33 | ,, | 3/6 |
| ,, | 256 | 83 | 24 | ,, | 0/6 |
| 2-Hexenoic lactone (V) | 256 | 79 | 15 | ,, | 1/7 |
| ,, | 256 | 83 | 7·5 | ,, | 0/6 |
| 4-Hexenoic lactone (VI) | 116 | 63 | 19 | Sarcoma | 0/6 |
| ,, | 116 | 72 | 13·5 | Fibrosarcoma | 2/6 |
| ,, | 116 | 95 | 13 | Sarcoma | 2/6 |
| Penicillin G (VII) (1670 i.u./mg.) | 184 | 59 | ? | Fibroma* | 0/6 |
| ditto | 184 | 72 | 5·5 | Fibrosarcoma | 0/6 |
| ditto | 208 | 84 | 13·5 | ,, | 5/6 |
| ditto | 184 | 100 | ? | Thyroid carcinoma* | NA |
| α-Carboxy-β-phenyl-β-propiolactone (VIII) | ? | 91 | 13 | Fibrosarcoma | 5/6 |
| αα-Diphenyl-β-propiolactone (IX) | 38 | 89 | 13 | Myxosarcoma | 0/5 |
| S-2-Carboxyethyl-L-cysteine (X) | 52 | 87 | 41 | Sarcoma | 1/6 |

NA—Not attempted.
*—Tumours which developed at sites remote from the injections.

cillin over a period of 46 weeks and at post mortem an enlarged lobe of the thyroid was found. Histological examination showed this to be a carcinoma of the epithelial cells of the thyroid follicles similar to the thyroid tumours which Lindsay, Potter and Chaikoff (1957) have reported to be of spontaneous origin in about 33 per cent of untreated animals examined by them. In contrast to this we have dissected out the thyroids of some hundreds of rats from our colony varying in age from 6 months to well over one year and have never noticed a similar condition. We rarely have the opportunity to examine the thyroids of rats which are 2 years old as was this penicillin treated rat. In this series of experiments, however, over 50 rats were examined which were more than 20 months old and in this selection no other thyroid tumours were found. Whether the thyroid tumour resulted from the penicillin treatment or not it is therefore impossible to say.

There were no tumours found in the oil-injected controls examined at 54 and 61 weeks after the treatment commenced. Among five rats which survived for more than 95 weeks after the oil injections were started there were no tumours at the site of the injections, but one of three rats which survived 107 weeks was found to have a dark red fleshy nodule inside the thoracic cavity, attached to the costal muscle layer, and lying to the right side of the vertebral column at the level of the thoracic vertebrae 5–6–7. This nodule appears to be malignant in that it is not encapsulated and has infiltrated local adipose tissue. Its cells resemble parathyroid tumour, but are not typical of functioning parathyroid cells. Again, a tumour of this type has not been detected in any other rats which have been examined in this laboratory.

Other abnormalities found were : the presence of pyelonephritis in the kidneys of two rats treated respectively with 1 mg. of the 4-hexenoic lactone twice weekly for 58 weeks and 2 mg. methyl protoanemonin twice weekly for 64 weeks. Both these rats were examined after the experiment had continued for about 100 weeks.

One rat treated with 2 mg. of the 3-hexenolactone twice weekly for 64 weeks and examined 41 weeks later showed evidence of peritonitis with adhesions between the intestine and the abdominal wall, extreme bronchiectasis with hardly any of the lungs capable of ventilation, accompanied by the presence of pleural fluid in the thorax. The auricles were much enlarged. Also one adrenal was 3 or 4 times larger than normal with a swollen adrenal artery. Histological examination of this gland showed a picture suggestive of medullary phaeochromocytoma.

Gillman, Gilbert and Spence (1953) report that in rats reared from animals derived from the Wistar Institute phaeochromocytomas were found in 76 per cent females and 85·5 per cent males aged 25 months or more. The incidence in other strains of rats is often not nearly so high, but it is very possible that the phaeochromocytoma found in this study was spontaneous and not related to the treatment with 3-hexenolactone. The same authors report an incidence of 14 thyroid carcinomas in 327 rats aged 13–32 months which further suggests that the thyroid carcinomas also arose spontaneously in our penicillin treated group.

### *Chemical Properties of the Lactones Used*

### *Condensation of β-propiolactone with cysteine*

L-Cysteine hydrochloride (3·14 g., 0·02 moles) was dissolved in 40 ml. water, neutralized to bromothymol blue by sodium hydroxide (9·6 ml. 2 N), and stirred with a solution of β-propiolactone (1·44 g., 0·02 mole, redistilled) in 20 ml. water.

The mixture became acidic and was titrated with 2 N NaOH at frequent intervals to maintain neutrality : 50 per cent of the calculated amount was required after 7 minutes at 20°, and 92 per cent after 2·5 hours, when hydrochloric acid (10 ml. 2 N) was added and the filtered solution was concentrated *in vacuo* until crystals appeared. After leaving at +4° overnight the crystalline compound was collected by filtration, washed with a little water and dried *in vacuo*. Total yield 2·88 g. nearly pure material, m.p. 196° decomp., or 73 per cent of calculated. After one recrystallization from a little hot water, 2·45 g. material (A) m.p. 210–212° uncorrected, decomp., was obtained. Analysis (Weiler and Strauss, Oxford) : Found : C, 37·0 ; H, 5·7 ; N, 7·27 ; S, 16·1 per cent. Calculated for $C_6H_{11}O_4NS$ : C, 37·2 ; H, 5·7 ; N, 7·35 ; S, 16·6 per cent. The formula is that of *S*-2-carboxyethyl-L-cysteine, of which a sample (B) kindly supplied by Dr. P. Mamalis (Mamalis *et al.*, 1960) had m.p. (uncorr.) 210–212° (decomp.) ; mixed m.p. unchanged. The N-benzoyl derivative was prepared by treatment with benzoyl chloride in alkali and had m.p. 103–105° (Mamalis *et al.*, 1960, give 106·5–108°), mixed m.p. 103–105°. Analysis (Weiler and Strauss) ; Found : C, 52·8 ; H, 5·22 ; N, 4·84 per cent. $C_{13}H_{15}O_5NS$ requires C, 52·8 ; H, 5·1 ; N, 4·7 per cent. The colour value of the unbenzoylated product with ninhydrin, after heating at 100° for 15 minutes, was per mole 90 per cent of that given by pure leucine, indicating one free α-amino group in the molecule. The nitroprusside test for thiol group was negative. On paper chromatography, both before and after oxidation (? to the sulphoxides) with dilute hydrogen peroxide, and whether run in phenol/water or butanol/acetic acid, the $R_F$ values were virtually identical with those obtained with the sample of *S*-2-carboxyethyl-L-cysteine of Mamalis *et al.* (1960) :

| | $R_F$ in : | |
|---|---|---|
| | Phenol/water | BuOH/acetic |
| β-Propiolactone product (A) . | 0·41 | 0·44 |
| *S*-Carboxyethyl cysteine (B) . | 0·41 | 0·46 |
| Oxidized product (A) . . | 0·27 | 0·26 |
| Oxidized product (B) . . | 0·27 | 0·26 |

We are greatly indebted to Dr. J. K. Whitehead for these measurements and for assistance in identification of our product.

Consequently the reaction of β-propiolactone with cysteine may be written :

where R— is HOOC . CH($NH_2$) . $CH_2$— of cysteine, and R . S—H the mercaptide ion.

*Rates of hydrolysis of the lactones*

The rates of hydrolysis were measured :

(*a*) In bicarbonate buffer at 37° and pH approximately 7 in Warburg manometers in an atmosphere of nitrogen containing 5 per cent carbon dioxide.

(*b*) By titration at 25° with carbon dioxide-free sodium hydroxide and phenolphthalein as indicator.

*Rates of reaction of the lactones with aqueous cysteine at approximately neutral reaction*

These rates were measured :

(*a*) By mixing, from the side bulb of a Warburg vessel, the lactone (usually 20 $\mu$moles) with a solution of cysteine (20 $\mu$moles) in 0·025 M sodium bicarbonate at 25° under nitrogen/5 per cent $CO_2$, and measuring the acid production manometrically as the $CO_2$ evolved.

(*b*) By measurement of free sulphydryl in a mixture, as under (*a*), by means of N-ethyl maleimide (Roberts and Ronser, 1958).

(*c*) By a similar reaction but with the use of the method of Sullivan, Hess and Howard (1942) for cysteine estimation.

This variety of methods was necessary because the lactones variously interfered with the colour reactions and with several of them the nitroprusside test, for example, was brown instead of purple and the results were completely unreliable.

The approximate relative rates of hydrolysis and of reaction with the sulphydryl group of cysteine are summarized in Table V.

TABLE V.—*Rates of Hydrolysis and of Reaction with Cysteine of the Lactones*

| | Lactone | Rate of hydrolysis | Rate of reaction with cysteine: Acid production | Loss of SH |
|---|---|---|---|---|
| I | β-Propiolactone | +++[a] | +++[c] | +++ |
| II | Patulin | − | ++++ | ++[e] |
| III | Penicillic acid | instantaneous[b] | ?[b] | ++[e] |
| IV | Methyl protoanemonin | ± | ++ | + |
| V | 4-Hexenoic lactone | ± | ++ | ++ |
| VI | 2-Hexenoic lactone | − | − | + |
| VII | Penicillin G | ± | +++[d] | + |
| XI | 3-Hexenoic lactone | + | +++ | ± |
| XII | α-Angelica lactone | ± | +++ | ± |
| XIII | γ-Butyrolactone | − | − | ± |

*a.* $k$ (unimolecular) at 25° : found = $3 \cdot 9 \times 10^{-3}$ min$^{-1}$.
*b.* The lactone is in tautomeric equilibrium with the corresponding ketoacid, 4-keto-3-methoxy-5-methylene-hex-2-enoic acid.
*c.* $k$ (bimolecular) at 25° : found = 4·6 lit. mole$^{-1}$. min$^{-1}$.
*d.* $k$ (bimolecular) at 23° is 4·9 lit. mole$^{-1}$. min$^{-1}$. (strongly pH-dependent ; Nakken *et al.*, 1960).
*e.* See Geiger and Conn (1945).

### DISCUSSION

As far as we are aware only β-propiolactone among the present series of compounds has hitherto been shown to be carcinogenic. In fact, the only other member of this series previously tested appears to be penicillin, of which various derivatives are cited as giving no tumours in the survey by Shubik and Hartwell (1957), where they appear under reference numbers 755, 768, 792, 895 and 910. Most of these penicillin derivatives were given orally, however, the longest administration being orally for 32 weeks in chicks dosed with procaine-penicillin G (Elam, Gee and Couch, 1951) and for 29 weeks in rats with *N*,*N*′-dibenzylethylene-diamine penicillin (Forbes *et al.*, 1953). The longest series of subcutaneous injections cited is only 7 weeks, a period almost certainly inadequate to elicit a carcinogenic response in the rats used, especially as an aqueous solution (of benzyl penicillin $\beta$-diethylaminoethyl ester hydriodide) was injected (Engelbreth-Holm and Roholt, 1953).

In the present series of experiments the control rats given arachis oil did not develop local tumours. The oil was unheated before use, unlike that used for similar control injections into rats by Walpole *et al.* (1954), which was sterilized by heating at 140° C. for 1 hour before use. These authors observed in 91 rats so injected, 14 sarcomas at the injection site after periods of from 60–100 weeks. Whether the difference is related to the pre-heating of the oil or not is unknown. In our control series also, only one tumour at a site remote from the injection was detected.

Local tumours were produced by injection of oily solutions of substances I–X, Tables I–IV. Without tests at higher dilutions it is not possible to attempt more than a very provisional grading of carcinogenic activity, but this would be approximately in descending order down the series from I to X. $\beta$-Propiolactone (I) was the most active, in that all survivors at a dosage of 0·1 mg. per injection developed local sarcomas within 34 weeks. It is of interest that even in freshly prepared aqueous solution, in which form it is fairly readily hydrolysed, $\beta$-propiolactone was also carcinogenic. Patulin (II) in doses of 0·2 mg. in oil produced local sarcomas in 6 rats among 8 survivors, after a longer period (64–69 weeks). Penicillic acid (III) was not tested in doses below 1 mg., at which level it produced tumours in all animals alive at 67 weeks. The activity of the three lactones, methyl protoanemonin (IV) the 2- and 4-hexenolactones (V and VI) appear to be roughly in that order, with penicillin G (VII) and the two substituted $\beta$-propiolactones (VIII and IX) rather less carcinogenically active in the doses given. Finally it was surprising to find one sarcoma in the series of rats injected with doses of 0·5 mg. of the condensation product of $\beta$-propiolactone and cysteine, namely *S*-2-carboxyethyl-L-cysteine (X) : injected rats dying earlier (47, 52, 69 and 76 weeks after start of injections) had no tumours. We are at present testing $\beta$-propiolactone hydrolysed by long standing in aqueous solution at 25° to 3-hydroxypropionic acid, but as yet (45 weeks) no tumours have developed in rats given 1 mg. doses of this compound. Painting $\beta$-bromopropionic acid on mouse skin (*Ann. Rep. Brit. Emp. Cancer Campgn*, 1959) gave no tumours either. There is perhaps a possibility that the carboxyethyl radicle may become transferred in the body from compound (X), a point that requires investigation.

Closely related lactones which have not produced tumours in this series are the saturated compound $\gamma$-butyrolactone (XIII) and two lactones both having the double bond in the $\beta$-position, namely the 3-hexenolactone (XI) and $\alpha$-angelica lactone (XII).

As a result of this work, the types of chemical structures shown in Table VI have been provisionally allocated potentially carcinogenic or non-carcinogenic properties :

The Roman figures under the formulae refer to the numbers given to the representative lactones in this paper, while the capital letters refer to the group-formulae in Table VI.

The 4-membered rings of the propiolactones (A) or of penicillin (B) ; and also the various hexenolactones (C, D and E) can be carcinogenic, but not apparently the saturated 5-membered lactone (F) nor the $\beta$-unsaturated lactones (G).

The carcinogenic activity in these series bears no obvious relationship to the rate of hydrolysis (Table V), but there may be a tentative indication of a cor-

TABLE VI.—*Basic Types of Chemical Structure in Relation to Carcinogenicity*

(a) Carcinogenic

A

Compounds I, VIII, IX

B

VII

C

Compounds II, III, IV, V

D

II, IV

E

VI

(b) Not carcinogenic

F

Compounds XIII

G

XI, XII

relation with the reactivity with the cysteine sulphydryl group, though apparently not with the extent of acid production occurring on reaction with cysteine (Table V). It would appear desirable to make a more detailed study of the mechanism of reaction of all these lactones with nucleophilic reagents such as cysteine, since the overall reactions clearly belong to more than one fundamental type. It is worth noting that among the five-membered lactones, the possibility of conjugation of the carbonyl double bond with other double bonds already present in the molecule (lactones II, III, IV, VI) or perhaps readily produced by dehydrogenation (for example of lactone V, giving the doubly conjugated lactone IV), appears to be associated with carcinogenic activity in this series of compounds.

A very wide range of such lactones is available, and many of them have been found to be naturally occurring in plants, bacteria and animals (see Haynes, 1948). Although all the compounds found to be carcinogenic in this paper are weakly so, relative to such highly active carcinogens as benzopyrene, Kennaway (1954) and others have stressed the importance in relation to human cancer which materials of low potency might have if they were to be either formed in animal metabolism or taken up from external sources over long periods.

The group of lactones and lactams of the type described in this paper need much further study with a view to developing the relationship between chemical structure and carcinogenesis in this series, which seems to be emerging. This should be supplemented by a study of the effects of the lactones as antibiotics, on mutations, as alkylating agents, and as selective growth inhibitors ; properties which as we have already seen are known to be possessed by some of their members.

### SUMMARY

1. Starting from the known carcinogenic property of β-propiolactone, a study has been made of the effect of twice weekly subcutaneous injection into rats of a number of chemically and pharmacologically active lactones and related substances.

2. β-Propiolactone given as a solution in arachis oil was found to produce sarcomas at the site of injection in all rats surviving at 34 weeks at a dose level of 0·1 mg.—about one twentieth of that previously tested. Tested at the 2 mg. dose level the freshly prepared aqueous solution was also carcinogenic. Two substituted β-propiolactones were weakly carcinogenic in the doses used.

3. The antibiotics patulin (clavacin) and penicillic acid were carcinogenic in all surviving rats when tested in 0·2 mg. and 2 mg. doses respectively.

4. Penicillin G (2 mg. doses as the sodium salt) suspended in arachis oil produced one local sarcoma in each of two separate groups of five rats.

5. Arachis oil, unheated, gave no local tumours in 20 control rats, which showed only one tumour (thoracic) at autopsy.

6. Three synthetic unsaturated 5-membered lactones (methyl protoanemonin, 2-hexenoic acid lactone and 4-hexenoic acid lactone), in doses of 1 or 2 mg., produced local sarcomas on injection.

7. Attention is drawn to the properties of antibiotic action and of growth inhibition, and their inactivation by reaction with cysteine, shown by certain of this group of carcinogenic agents. The interaction of β-propiolactone and cysteine has been studied and the product isolated and identified as *S*-2-carboxyethyl-L-cysteine. This substance itself had only very weak carcinogenic properties.

8. Two main types of chemical constitution appear associated with carcinogenic activity in this series : a highly strained 4-membered ring (as in the β-propiolactones and the lactam ring in penicillin) or a lactone ring having double bonds at the 2 or 4 position, or preferably at both. If there was no double bond, or if it was at the 3-position, the lactones tested were inactive.

We would like to express our gratitude to Dr. A. C. Thackray for his kindness in giving opinions on all the sections examined histologically, and also to Professor I. Doniach for advice on the thyroid and (?)parathyroid tumours. Gifts of materials from Professor J. H. Birkinshaw, Dr. P. Mamalis, Professor H. Oettel and Dr. A. L. Walpole are gratefully acknowledged. Dr. J. K. Whitehead and Dr. A. E. Kellie and Mr. D. H. Williamson kindly provided chemical advice and help, and technical assistance was provided by Mr. S. Graves, Mr. E. D. Decker, Miss Frances Bell and Miss Judith Cooke.

This work was supported by a block grant made to the Medical School by the British Empire Cancer Campaign.

### REFERENCES

Annual Report of the British Empire Cancer Campaign.—(1959) Report of the Birmingham Branch, **37**, 371.

Bartlett, P. D. and Small, G., Jr.—(1950) *J. Amer. chem. Soc.*, **72**, 4867.

Bernheim, F. and Gale, G. R.—(1952) *Proc. Soc. exp. Biol., N.Y.*, **80**, 162.

Birkinshaw, J. H., Oxford, A. E. and Raistrick, H.—(1936) *Biochem. J.*, **30**, 394.

Cavallito, C. J. and Haskell, T. H.—(1945) *J. Amer. chem. Soc.*, **67**, 1991.

Dickens, F., Jones, H. E. H. and Williamson, D. H.—(1956) *Rep. Brit. Emp. Cancer Campgn.*, **34**, 100.

Elam, J. F., Gee, L. L. and Couch, J. R.—(1951) *Proc. Soc. exp. Biol., N.Y.*, **77**, 209.

Engelbreth-Holm, J. and Roholt, K.—(1953) *Acta pharm. tox., Kbh.*, **9**, 117.

Forbes, M., Shockman, G. D., Chu, E. and Gyergy, P.—(1953) *Antibiotics and Chemother.*, **3**, 1104.

Friedewald, W. F. and Rous, P.—(1944) *J. exp. Med.*, **80**, 101.

Gale, G. R.—(1953) *J. Bact.*, **65**, 505.

Geiger, W. B. and Conn, J. E.—(1945) *J. Amer. chem. Soc.*, **67**, 112.

Gillman, J., Gilbert, C. and Spence, I.—(1953) *Cancer*, **6**, 494.

Gresham, T. L., Jansen, J. E., Shaver, F. W. and Gregory, J. T.—(1948) *J. Amer. chem. Soc.*, **70**, 999.

Hartman, F. W., LoGrippo, G. and Kelly, A. R.—(1954) *Amer. J. clin. Path.*, **24**, 399.

Hartwell, J. L.—(1951) 'Survey of Compounds which have been tested for Carcinogenic Activity' (2nd Ed.). Washington, D.C. (U.S. Public Health Service.)

Hauschka, T. S., Toennies, G. and Swain, A. R.—(1945) *Science*, **101**, 383.

Haynes, L. J.—(1948) *Quart. Rev. chem. Soc., Lond.*, **2**, 46.

Heaton, T. B.—(1929) *J. Path. Bact.*, **32**, 565.

Kennaway, E. L.—(1954) *Brit. med. J.*, ii, 663.

Kühn, R. and Jerchel, D.—(1943) *Chem. Ber.*, **76**, 413.

*Iidem*, Moewus, F., Möller, E. F. and Lettre, H.—(1943) *Naturwissenschaften*, **31**, 468.

Lindsay, S., Potter, G. D. and Chaikoff, I. L.—(1957) *Cancer Res.*, **17**, 183.

Mamalis, P., McHale, D. and Green, J.—(1960) *J. chem. Soc.*, 2906.

Medawar, P. B.—(1937) *Quart. J. exp. Physiol.*, **27**, 147.

*Idem*, Robinson, G. M. and Robinson, R.—(1943) *Nature, Lond.*, **151**, 195.

Nakken, K. F., Eldjarn, L. and Pihl, A.—(1960) *Biochem. Pharmacol.*, **3**, 89.

Orlans, E. S. and Jones, V. E.—(1958) *Nature, Lond.*, **182**, 1216.

Rains, A. J. H., Crawford, N., Sharpe, S. H., Shrewsbury, J. F. D. and Barson, G. J.—(1956) *Lancet*, ii, 830.

Roberts, A. and Ronser, M. M.—(1958) *Analyt. Chem.*, **30**, 1291.

Roe, F. J. C. and Glendenning, O. M.—(1956) *Brit. J. Cancer*, **10**, 357.

*Idem* and Salaman, M. H.—(1955) *Ibid.*, **9**, 177.—(1958) *Brit. med. J.*, i, 942.

Ross, W. C. J.—(1953) *In : Advanc. Cancer Res.*, **1**, 397. New York (Academic Press).

Salaman, M. H.—(1959) *In : Ciba Foundation Symposium on Carcinogenesis.* Ed. by G. E. W. Wolstenholme and M. O'Connor. London (J. & A. Churchill), p. 70.

Shubik, P. and Hartwell, J. L.—(1957) Supplement I to 'Survey of Compounds which have been Tested for Carcinogenic Activity'. Washington, D.C. (U.S. Public Health Service.)

Smith, H. H. and Srb, A. M.—(1951) *Science*, **114**, 490.

Sullivan, M. X., Hess, W. C. and Howard, H. W.—(1942) *J. biol. Chem.*, **145**, 621.

Walpole, A. L., Roberts, D. C., Rose, F. L., Hendry, J. A. and Homer, R. F.—(1954) *Brit. J. Pharmacol.*, **9**, 306.